# 2-Ethyl-3-[(*R*)-2-phenyl­butanamido]­quinazolin-4(3*H*)-one monohydrate

**DOI:** 10.1107/S1600536814005996

**Published:** 2014-03-22

**Authors:** Gamal A. El-Hiti, Keith Smith, Amany S. Hegazy, D. Heulyn Jones, Benson M. Kariuki

**Affiliations:** a Cornea Research Chair, Department of Optometry, College of Applied Medical Sciences, King Saud University, PO Box 10219, Riyadh 11433, Saudi Arabia; b School of Chemistry, Cardiff University, Main Building, Park Place, Cardiff CF10 3AT, Wales

## Abstract

In the title compound, C_20_H_21_N_3_O_2_·H_2_O (EQR·H_2_O), the quinazoline ring system forms dihedral angles of 53.1 (1) and 85.6 (1)° with the phenyl ring and the amide link, respectively. In the crystal, O—H⋯O hydrogen bonds link two EQR and two water mol­ecules into a centrosymmetric *R*_4_^4^(18) ring motif. N—H⋯O hydrogen bonds further link these hydrogen-bonded fragments into columns extending in [010].

## Related literature   

For convenient routes towards modifying 3*H*-quinazolin-4-one derivatives, see: Smith *et al.* (1995 ▶, 1996*a* ▶,*b* ▶, 2004 ▶). For the crystal structures of related compounds, see: Yang *et al.* (2009 ▶); Srinivasan *et al.* (2011 ▶).
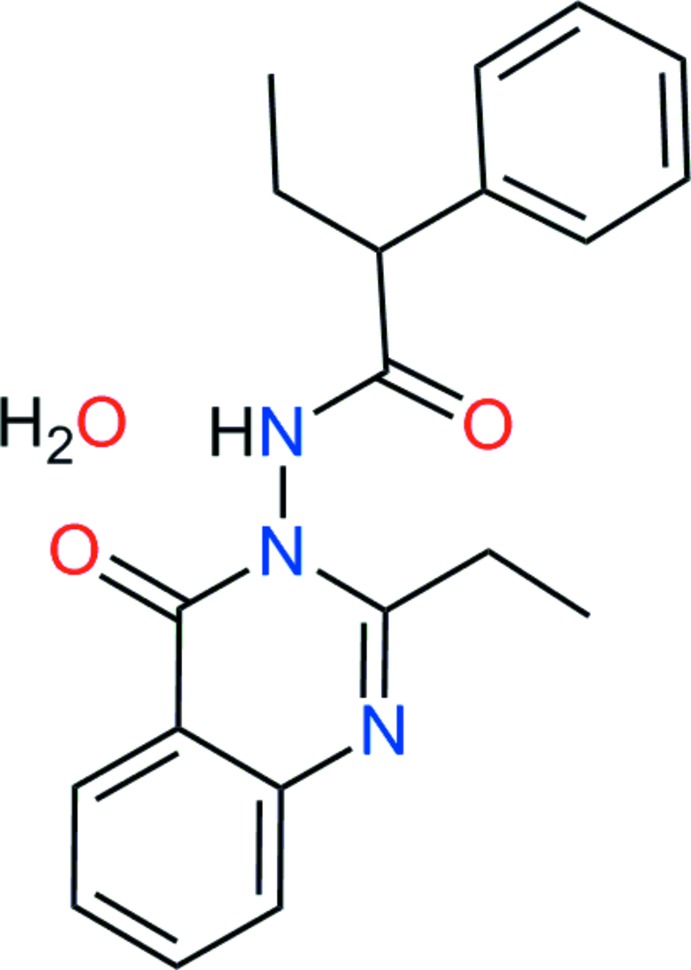


## Experimental   

### 

#### Crystal data  

C_20_H_21_N_3_O_2_·H_2_O
*M* *_r_* = 353.41
Monoclinic, 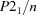

*a* = 14.5354 (2) Å
*b* = 7.3529 (1) Å
*c* = 18.1945 (3) Å
β = 98.591 (1)°
*V* = 1922.76 (5) Å^3^
*Z* = 4
Cu *K*α radiation
μ = 0.68 mm^−1^
*T* = 296 K
0.41 × 0.21 × 0.08 mm

#### Data collection  

Agilent SuperNova (Dual, Cu at zero, Atlas) diffractometer
Absorption correction: gaussian (*CrysAlis PRO*; Agilent, 2014 ▶) *T*_min_ = 0.723, *T*_max_ = 1.000
13383 measured reflections
3796 independent reflections
3341 reflections with *I* > 2σ(*I*)
*R*_int_ = 0.017

#### Refinement  

*R*[*F* ^2^ > 2σ(*F* ^2^)] = 0.040
*wR*(*F* ^2^) = 0.116
*S* = 1.05
3796 reflections
246 parameters
H atoms treated by a mixture of independent and constrained refinement
Δρ_max_ = 0.29 e Å^−3^
Δρ_min_ = −0.36 e Å^−3^

### 

Data collection: *CrysAlis PRO* (Agilent, 2014 ▶); cell refinement: *CrysAlis PRO*; data reduction: *CrysAlis PRO*; program(s) used to solve structure: *SHELXS2013* (Sheldrick, 2008 ▶); program(s) used to refine structure: *SHELXL2013* (Sheldrick, 2008 ▶); molecular graphics: *ORTEP-3 for Windows* (Farrugia, 2012 ▶); software used to prepare material for publication: *WinGX* (Farrugia, 2012 ▶) and *CHEMDRAW Ultra* (CambridgeSoft, 2001 ▶).

## Supplementary Material

Crystal structure: contains datablock(s) I, New_Global_Publ_Block. DOI: 10.1107/S1600536814005996/cv5446sup1.cif

Structure factors: contains datablock(s) I. DOI: 10.1107/S1600536814005996/cv5446Isup2.hkl

Supporting information file. DOI: 10.1107/S1600536814005996/cv5446Isup3.cml

CCDC reference: 992297

Additional supporting information:  crystallographic information; 3D view; checkCIF report

## Figures and Tables

**Table 1:** Hydrogen-bond geometry (Å, °)

*D*—H⋯*A*	*D*—H	H⋯*A*	*D*⋯*A*	*D*—H⋯*A*
N3—H3⋯O3	0.86	1.92	2.7431 (15)	159
O3—H3*A*⋯O2^i^	0.92 (3)	1.89 (3)	2.7806 (15)	164 (2)
O3—H3*B*⋯O1^ii^	0.91 (3)	1.92 (3)	2.8154 (17)	169 (2)

Symmetry codes: (i) 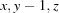; (ii) 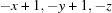.

**Fig. 1.**
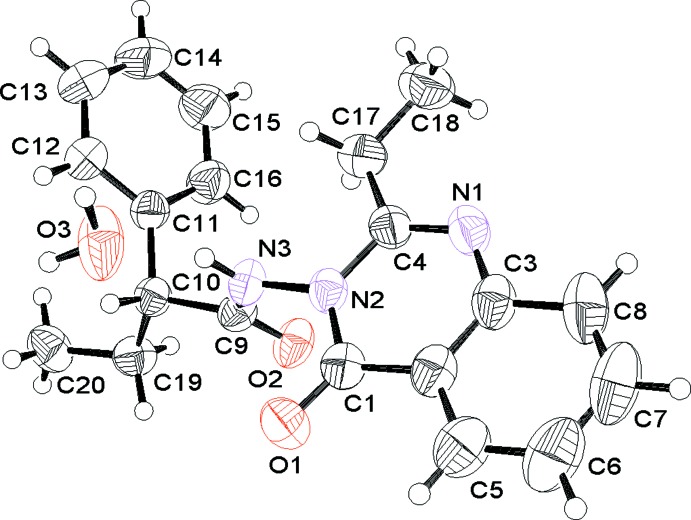
The molecular structure of the title compound showing the atomic numbering and 50% probability displacement ellipsoids for non-H atoms.

**Fig. 2.**
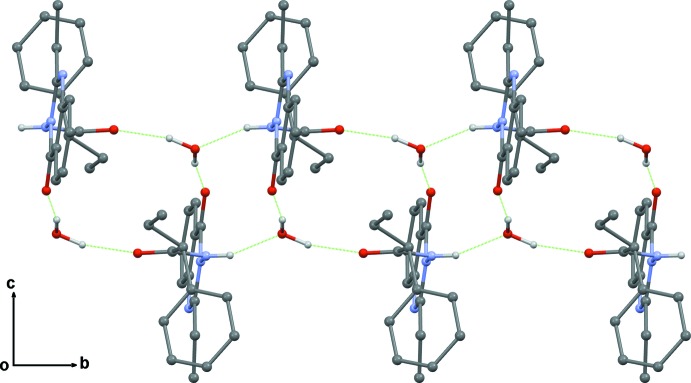
A portion of the crystal packing viewed along the a axis and showing the hydrogen bonds as dotted green lines. C-bound H atoms were omitted for clarity.

## supplementary crystallographic information

### 1. Comment

In a continuation of our research focused on new synthetic routes towards novel substituted 3*H*-quinazolin-4-one derivatives (Smith *et al.*, 1995, 1996**a*,b*, 2004) we have synthesized 2-ethyl-3-((*R*)-2-phenylbutanoylamino)-3*H*-quinazolin-4-one (EQR) in a high yield. Herewith we present the crystal structure of its monohydrate.

In the title compound, EQR.H_2_O (Fig. 1), all bond lengths and angles are normal and correspond well to those observed in the related structures (Smith *et al.*, 2004; Yang *et al.*, 2009; Srinivasan *et al.*, 2011). The quinazoline ring system forms a dihedral angle of 53.1 (1)° with the phenyl ring and an angle of 85.6 (1)° with the amide link. A pair of EQR molecules accepts O–H···O hydrogen bonds from two water molecules to form a R^4^_4_(18) ring motif. These rings are linked by N–H···O bonds to form columns parallel to the b-axis (Fig. 2).

### 2. Experimental

To a stirred mixture of 3-amino-2-ethyl-3*H*-quinazolin-4-one (1.89 g, 10.0 mmol) and Et_3_N (3 ml) in anhydrous toluene (20 ml), was added a solution of 2-phenylbutanoyl chloride (0.91 g, 5.0 mmol) in anhydrous toluene (5 ml). The mixture was heated under reflux for 30 min, allowed to cool, washed with saturated aqueous NaHCO_3_ (2 x 10 ml) and H_2_O (2 x 15 ml), dried (MgSO_4_), and evaporated under reduced pressure. The residue obtained was purified by column chromatography on silica gel (Et_2_O–hexane, 1:4) followed by recrystallization from ethyl acetate to give 2-ethyl-3-(2-phenylbutanoylamino)-3*H*-quinazolin-4-one (1.36 g, 4.05 mmol; 81% based on acid chloride) as colourless crystals, m.p. 101–103 °C. The title compound (I) appears from its NMR spectra as a mixture of two diastereoisomers in unequal proportions (Ia:Ib = *ca*. 4:6), due to restricted rotation around the *N*–*N* axis (Smith *et al.*, 2004), but the X-ray crystallography showed a single type of crystal containing just one diastereoisomer but with both enantiomers in equal proportions (the structure displayed shows the structure as 2-ethyl-3-((*R*)-2-phenylbutanoylamino)-3*H*-quinazolin-4-one hydrate). The ^1^H NMR spectrum also showed diastereotopism for the CH_2_ protons of the ethyl groups, but was temperature dependent and showed a single set of signals at 150 °C (Smith *et al.*, 2004). EI–MS: *m/z* (%) = 335 (*M*^+^, 5), 216 (40), 189 (17), 173 (12), 119 (65), 91 (100). HRMS (EI): Calculated for C_20_H_21_N_3_O_2_ [*M*]: 335.1634; found, 335.1634. NMR assignments have been made on the basis of expected chemical shifts and coupling patterns and have not been rigorously confirmed.

^1^H NMR (500 MHz, DMSO-*d*_6_, *δ*, p.p.m.) 8.15 (d, *J* = 8 Hz, 0.6 H, H-5 of Ib), 8.06 (d, *J* = 8 Hz, 0.4 H, H-5 of Ia), 7.87–7.82 (m, 1 H, H-7 of Ia and Ib), 7.68 (d, *J* = 8 Hz, 0.4 H, H-8 of Ia), 7.65 (d, *J* = 8 Hz, 0.6 H, H-8 of Ib), 7.57–5.48 (m, 1 H, H-6 of Ia and Ib), 7.45–7.35 (m, 4 H, H-2 and H-3 of Ph of Ia and Ib), 7.32–7.27 (m, 1 H, H-4 of Ph of Ia and Ib), 3.75–3.67 (m, 1 H, CH of Ia and Ib), 2.76 (dq, *J* = 15, 7.5 Hz, 0.4 H, ArC*H_a_*H_b_ of Ia), 2.63 (dq, *J* = 15, 7.5 Hz, 0.4 H, ArCH_a_*H_b_* of Ia), 2.30 (dq, *J* = 15, 7.5 Hz, 0.6 Hz, ArC*H_a_*H_b_ of Ib), 2.20 (dq, *J* = 15, 7.5 Hz, 0.6 H, ArCH_a_*H_b_* of Ib), 2.18–2.04 (m, 1 H, CHC*H_a_*H_b_ of Ia and Ib), 1.86–1.71 (m, 1 H, CHCH_a_*H_b_* of Ia and Ib), 1.25 (app. t, *J* = 7.5 Hz, 1.2 H, C*H_3_*CH_2_Ar of Ia), 0.99 (app. t, *J* = 7.5 Hz, 1.8 H, C*H_3_*CH_2_Ar of Ib), 0.96–0.90 (m, 3 H, C*H_3_*CH_2_CH of Ia and Ib). ^13^C NMR (125 MHz, DMSO-*d*_6_, *δ*, p.p.m.) 173.0 (s, C=O of Ia), 172.9 (s, C=O of Ib), 159.7 (s, C-4 of Ib), 159.6 (s, C-4 of Ia), 159.5 (s, C-2 of Ib), 159.3 (s, C-2 of Ia), 147.1 (s, C-8a of Ib), 147.0 (s, C-8a of Ia), 139.9 (s, C-1 of Ph of Ib), 139.6 (s, C-1 of Ph of Ia), 135.4 (d, C-7 of Ib), 135.3 (d, C-7 of Ia), 128.9 (d, C-3/C-5 of Ph of Ib), 128.7 (d, C-3/C-5 of Ph of Ia), 128.5 (d, C-2/C-6 of Ph od Ia), 128.1 (d, C-2/C-6 of Ph of Ib), 127.6 (d, three overlapping signals, C-6 of Ia and Ib and C-8 of Ib), 127.4 (d, C-8 of Ia), 127.2 (d, C-5 of Ib), 127.1 (d, C-5 of Ia), 126.9 (d, C-4 of Ph), 126.8 (d, C-4 of Ph), 121.1 (s, two overlapping signals, C-4a of Ia and Ib), 51.9 (d, CH of Ia), 51.8 (d, CH of Ib), 27.0 (t, *C*H_2_CH of Ia), 26.9 (t, *C*H_2_CH of Ib), 26.6 (t, CH_2_Ar of Ib), 25.9 (t, CH_2_Ar of Ia), 12.6 (q, *C*H_3_CH_2_CH of Ia), 12.5 (q, *C*H_3_CH_2_CH of Ib), 11.0 (q, *C*H_3_CH_2_Ar of Ia), 10.8 (q, *C*H_3_CH_2_Ar of Ib).

### 3. Refinement

The hydrogen atoms of the water molecule were located in the difference Fourier map and refined isotropically. The C- and N-bound hydrogen atoms were positioned geometrically, and refined using a riding model, with *U*_iso_(H) = 1.2–1.5 *U*_eq_ of the parent atom.

### Crystal data

**Table:**

C_20_H_21_N_3_O_2_·H_2_O	*F*(000) = 752
*M**_r_* = 353.41	*D*_x_ = 1.221 Mg m^−^^3^
Monoclinic, *P*2_1_/*n*	Cu *K*α radiation, λ = 1.54184 Å
*a* = 14.5354 (2) Å	Cell parameters from 3341 reflections
*b* = 7.3529 (1) Å	θ = 3.6–67.7°
*c* = 18.1945 (3) Å	µ = 0.68 mm^−^^1^
β = 98.591 (1)°	*T* = 296 K
*V* = 1922.76 (5) Å^3^	Plate, colourless
*Z* = 4	0.41 × 0.21 × 0.08 mm

### Data collection

**Table:**

Agilent SuperNova (Dual, Cu at zero, Atlas) diffractometer	3341 reflections with *I* > 2σ(*I*)
Radiation source: sealed X-ray tube	*R*_int_ = 0.017
ω scans	θ_max_ = 73.5°, θ_min_ = 3.6°
Absorption correction: gaussian (*CrysAlis PRO*; Agilent, 2014)	*h* = −16→17
*T*_min_ = 0.723, *T*_max_ = 1.000	*k* = −9→8
13383 measured reflections	*l* = −22→21
3796 independent reflections	

### Refinement

**Table:**

Refinement on *F*^2^	Hydrogen site location: mixed
Least-squares matrix: full	H atoms treated by a mixture of independent and constrained refinement
*R*[*F*^2^ > 2σ(*F*^2^)] = 0.040	*w* = 1/[σ^2^(*F*_o_^2^) + (0.0544*P*)^2^ + 0.3851*P*] where *P* = (*F*_o_^2^ + 2*F*_c_^2^)/3
*wR*(*F*^2^) = 0.116	(Δ/σ)_max_ < 0.001
*S* = 1.05	Δρ_max_ = 0.29 e Å^−^^3^
3796 reflections	Δρ_min_ = −0.36 e Å^−^^3^
246 parameters	Extinction correction: *SHELXL2013* (Sheldrick, 2008), Fc^*^=kFc[1+0.001xFc^2^λ^3^/sin(2θ)]^-1/4^
0 restraints	Extinction coefficient: 0.0033 (3)

### Special details

**Table:**

Experimental. Absorption correction: CrysAlisPro (Agilent, 2014): Numerical absorption correction based on Gaussian integration over a multifaceted crystal model. Empirical absorption correction using spherical harmonics, implemented in SCALE3 ABSPACK scaling algorithm.
Geometry. All e.s.d.'s (except the e.s.d. in the dihedral angle between two l.s. planes) are estimated using the full covariance matrix. The cell e.s.d.'s are taken into account individually in the estimation of e.s.d.'s in distances, angles and torsion angles; correlations between e.s.d.'s in cell parameters are only used when they are defined by crystal symmetry. An approximate (isotropic) treatment of cell e.s.d.'s is used for estimating e.s.d.'s involving l.s. planes.

### Fractional atomic coordinates and isotropic or equivalent isotropic displacement parameters (Å^2^)

**Table:**

	*x*	*y*	*z*	*U*_iso_*/*U*_eq_	
C1	0.71716 (9)	0.66803 (17)	0.06334 (7)	0.0466 (3)	
C2	0.81747 (9)	0.69215 (18)	0.07879 (8)	0.0525 (3)	
C3	0.85958 (9)	0.72040 (19)	0.15199 (9)	0.0557 (3)	
C4	0.72196 (9)	0.69526 (17)	0.19860 (7)	0.0470 (3)	
C5	0.87036 (12)	0.6905 (3)	0.02072 (11)	0.0732 (5)	
H5	0.8418	0.6688	−0.0277	0.088*	
C6	0.96387 (14)	0.7207 (3)	0.03496 (15)	0.0952 (7)	
H6	0.9992	0.7205	−0.0037	0.114*	
C7	1.00601 (13)	0.7517 (3)	0.10751 (16)	0.0979 (7)	
H7	1.0697	0.7731	0.1168	0.117*	
C8	0.95599 (11)	0.7514 (3)	0.16581 (13)	0.0796 (5)	
H8	0.9857	0.7715	0.2140	0.096*	
C9	0.52278 (8)	0.79347 (16)	0.10850 (7)	0.0410 (3)	
C10	0.41982 (8)	0.74708 (17)	0.10069 (7)	0.0418 (3)	
H10	0.4092	0.6357	0.0711	0.050*	
C11	0.39529 (8)	0.70884 (18)	0.17738 (7)	0.0456 (3)	
C12	0.34870 (10)	0.5513 (2)	0.19121 (8)	0.0593 (4)	
H12	0.3334	0.4666	0.1534	0.071*	
C13	0.32464 (13)	0.5187 (3)	0.26100 (10)	0.0781 (5)	
H13	0.2928	0.4128	0.2695	0.094*	
C14	0.34730 (15)	0.6408 (3)	0.31718 (10)	0.0835 (5)	
H14	0.3308	0.6185	0.3638	0.100*	
C15	0.39456 (14)	0.7967 (3)	0.30473 (9)	0.0769 (5)	
H15	0.4110	0.8791	0.3432	0.092*	
C16	0.41781 (11)	0.8314 (2)	0.23522 (8)	0.0600 (4)	
H16	0.4490	0.9383	0.2271	0.072*	
C17	0.66472 (11)	0.6937 (2)	0.26021 (8)	0.0611 (4)	
H17A	0.6266	0.5848	0.2559	0.073*	
H17B	0.6232	0.7977	0.2545	0.073*	
C18	0.72124 (14)	0.6992 (3)	0.33713 (9)	0.0749 (5)	
H18A	0.7599	0.5929	0.3445	0.112*	
H18B	0.6800	0.7021	0.3737	0.112*	
H18C	0.7597	0.8060	0.3419	0.112*	
C19	0.35937 (9)	0.8981 (2)	0.06048 (7)	0.0512 (3)	
H19A	0.3635	1.0057	0.0916	0.061*	
H19B	0.3826	0.9292	0.0148	0.061*	
C20	0.25831 (10)	0.8397 (2)	0.04242 (9)	0.0628 (4)	
H20A	0.2540	0.7337	0.0113	0.094*	
H20B	0.2224	0.9364	0.0169	0.094*	
H20C	0.2346	0.8119	0.0876	0.094*	
N1	0.81045 (8)	0.72053 (16)	0.21145 (7)	0.0557 (3)	
N2	0.67465 (7)	0.66873 (14)	0.12675 (6)	0.0429 (2)	
N3	0.57841 (7)	0.64529 (14)	0.11660 (6)	0.0446 (3)	
H3	0.5547	0.5380	0.1155	0.054*	
O1	0.67227 (7)	0.64818 (15)	0.00151 (5)	0.0613 (3)	
O2	0.55448 (6)	0.94696 (12)	0.11169 (6)	0.0557 (3)	
O3	0.50861 (9)	0.30699 (16)	0.07666 (10)	0.0873 (5)	
H3A	0.5189 (19)	0.193 (4)	0.0963 (14)	0.124 (9)*	
H3B	0.450 (2)	0.306 (4)	0.0514 (15)	0.123 (9)*	

### Atomic displacement parameters (Å^2^)

**Table:**

	*U*^11^	*U*^22^	*U*^33^	*U*^12^	*U*^13^	*U*^23^
C1	0.0444 (6)	0.0406 (6)	0.0543 (7)	0.0049 (5)	0.0061 (5)	0.0001 (5)
C2	0.0436 (7)	0.0456 (7)	0.0687 (8)	0.0041 (5)	0.0100 (6)	0.0023 (6)
C3	0.0412 (7)	0.0474 (7)	0.0766 (9)	0.0026 (5)	0.0032 (6)	0.0011 (6)
C4	0.0482 (7)	0.0384 (6)	0.0518 (7)	0.0015 (5)	−0.0008 (5)	0.0040 (5)
C5	0.0592 (9)	0.0781 (11)	0.0871 (12)	0.0042 (8)	0.0264 (8)	−0.0011 (9)
C6	0.0634 (11)	0.1038 (16)	0.1273 (19)	−0.0029 (10)	0.0433 (12)	−0.0105 (14)
C7	0.0429 (9)	0.1033 (15)	0.151 (2)	−0.0051 (9)	0.0268 (11)	−0.0150 (15)
C8	0.0432 (8)	0.0802 (12)	0.1115 (15)	−0.0009 (8)	−0.0010 (8)	−0.0100 (10)
C9	0.0421 (6)	0.0375 (6)	0.0439 (6)	0.0001 (5)	0.0077 (5)	0.0028 (5)
C10	0.0387 (6)	0.0418 (6)	0.0451 (6)	−0.0001 (5)	0.0069 (5)	0.0000 (5)
C11	0.0392 (6)	0.0504 (7)	0.0473 (6)	−0.0002 (5)	0.0070 (5)	0.0036 (5)
C12	0.0591 (8)	0.0608 (9)	0.0582 (8)	−0.0127 (7)	0.0088 (6)	0.0058 (7)
C13	0.0840 (12)	0.0812 (12)	0.0721 (10)	−0.0192 (9)	0.0212 (9)	0.0191 (9)
C14	0.1001 (14)	0.0986 (14)	0.0564 (9)	−0.0080 (11)	0.0265 (9)	0.0127 (9)
C15	0.0934 (13)	0.0861 (12)	0.0536 (9)	−0.0062 (10)	0.0185 (8)	−0.0089 (8)
C16	0.0655 (9)	0.0603 (9)	0.0561 (8)	−0.0090 (7)	0.0157 (7)	−0.0048 (6)
C17	0.0644 (9)	0.0651 (9)	0.0533 (8)	0.0004 (7)	0.0074 (6)	0.0071 (7)
C18	0.0978 (13)	0.0720 (11)	0.0522 (8)	−0.0035 (9)	0.0023 (8)	0.0017 (7)
C19	0.0465 (7)	0.0560 (8)	0.0516 (7)	0.0083 (6)	0.0096 (5)	0.0071 (6)
C20	0.0473 (7)	0.0793 (10)	0.0590 (8)	0.0111 (7)	−0.0012 (6)	−0.0030 (7)
N1	0.0464 (6)	0.0538 (7)	0.0627 (7)	0.0000 (5)	−0.0057 (5)	0.0015 (5)
N2	0.0356 (5)	0.0398 (5)	0.0521 (6)	0.0007 (4)	0.0025 (4)	0.0010 (4)
N3	0.0350 (5)	0.0363 (5)	0.0618 (6)	−0.0024 (4)	0.0046 (4)	0.0000 (4)
O1	0.0554 (6)	0.0734 (7)	0.0533 (6)	0.0040 (5)	0.0022 (4)	−0.0047 (5)
O2	0.0473 (5)	0.0367 (5)	0.0830 (7)	−0.0019 (4)	0.0092 (4)	0.0053 (4)
O3	0.0695 (8)	0.0397 (6)	0.1395 (13)	−0.0015 (5)	−0.0278 (8)	−0.0025 (6)

### Geometric parameters (Å, º)

**Table:**

C1—O1	1.2220 (16)	C12—C13	1.387 (2)
C1—N2	1.3879 (17)	C12—H12	0.9300
C1—C2	1.4539 (18)	C13—C14	1.364 (3)
C2—C3	1.396 (2)	C13—H13	0.9300
C2—C5	1.397 (2)	C14—C15	1.372 (3)
C3—N1	1.382 (2)	C14—H14	0.9300
C3—C8	1.405 (2)	C15—C16	1.381 (2)
C4—N1	1.2860 (17)	C15—H15	0.9300
C4—N2	1.3969 (16)	C16—H16	0.9300
C4—C17	1.493 (2)	C17—C18	1.514 (2)
C5—C6	1.363 (3)	C17—H17A	0.9700
C5—H5	0.9300	C17—H17B	0.9700
C6—C7	1.389 (3)	C18—H18A	0.9600
C6—H6	0.9300	C18—H18B	0.9600
C7—C8	1.373 (3)	C18—H18C	0.9600
C7—H7	0.9300	C19—C20	1.519 (2)
C8—H8	0.9300	C19—H19A	0.9700
C9—O2	1.2172 (15)	C19—H19B	0.9700
C9—N3	1.3516 (16)	C20—H20A	0.9600
C9—C10	1.5208 (16)	C20—H20B	0.9600
C10—C11	1.5171 (17)	C20—H20C	0.9600
C10—C19	1.5324 (17)	N2—N3	1.3941 (14)
C10—H10	0.9800	N3—H3	0.8600
C11—C12	1.3836 (19)	O3—H3A	0.92 (3)
C11—C16	1.387 (2)	O3—H3B	0.91 (3)
			
O1—C1—N2	121.53 (12)	C13—C14—C15	119.81 (16)
O1—C1—C2	125.08 (13)	C13—C14—H14	120.1
N2—C1—C2	113.39 (12)	C15—C14—H14	120.1
C3—C2—C5	120.71 (14)	C14—C15—C16	120.18 (17)
C3—C2—C1	119.13 (13)	C14—C15—H15	119.9
C5—C2—C1	120.14 (14)	C16—C15—H15	119.9
N1—C3—C2	122.86 (12)	C15—C16—C11	120.84 (15)
N1—C3—C8	118.55 (15)	C15—C16—H16	119.6
C2—C3—C8	118.58 (16)	C11—C16—H16	119.6
N1—C4—N2	121.97 (13)	C4—C17—C18	114.06 (14)
N1—C4—C17	121.28 (12)	C4—C17—H17A	108.7
N2—C4—C17	116.75 (11)	C18—C17—H17A	108.7
C6—C5—C2	120.05 (19)	C4—C17—H17B	108.7
C6—C5—H5	120.0	C18—C17—H17B	108.7
C2—C5—H5	120.0	H17A—C17—H17B	107.6
C5—C6—C7	119.54 (19)	C17—C18—H18A	109.5
C5—C6—H6	120.2	C17—C18—H18B	109.5
C7—C6—H6	120.2	H18A—C18—H18B	109.5
C8—C7—C6	121.67 (17)	C17—C18—H18C	109.5
C8—C7—H7	119.2	H18A—C18—H18C	109.5
C6—C7—H7	119.2	H18B—C18—H18C	109.5
C7—C8—C3	119.43 (19)	C20—C19—C10	111.44 (12)
C7—C8—H8	120.3	C20—C19—H19A	109.3
C3—C8—H8	120.3	C10—C19—H19A	109.3
O2—C9—N3	121.75 (11)	C20—C19—H19B	109.3
O2—C9—C10	124.94 (11)	C10—C19—H19B	109.3
N3—C9—C10	113.18 (10)	H19A—C19—H19B	108.0
C11—C10—C9	108.59 (10)	C19—C20—H20A	109.5
C11—C10—C19	112.12 (10)	C19—C20—H20B	109.5
C9—C10—C19	111.61 (10)	H20A—C20—H20B	109.5
C11—C10—H10	108.1	C19—C20—H20C	109.5
C9—C10—H10	108.1	H20A—C20—H20C	109.5
C19—C10—H10	108.1	H20B—C20—H20C	109.5
C12—C11—C16	118.18 (13)	C4—N1—C3	118.51 (12)
C12—C11—C10	120.74 (12)	C1—N2—N3	116.87 (10)
C16—C11—C10	121.08 (12)	C1—N2—C4	124.09 (11)
C11—C12—C13	120.56 (15)	N3—N2—C4	119.02 (10)
C11—C12—H12	119.7	C9—N3—N2	119.14 (10)
C13—C12—H12	119.7	C9—N3—H3	120.4
C14—C13—C12	120.43 (16)	N2—N3—H3	120.4
C14—C13—H13	119.8	H3A—O3—H3B	106 (2)
C12—C13—H13	119.8		

### Hydrogen-bond geometry (Å, º)

**Table:**

*D*—H···*A*	*D*—H	H···*A*	*D*···*A*	*D*—H···*A*
N3—H3···O3	0.86	1.92	2.7431 (15)	159
O3—H3*A*···O2^i^	0.92 (3)	1.89 (3)	2.7806 (15)	164 (2)
O3—H3*B*···O1^ii^	0.91 (3)	1.92 (3)	2.8154 (17)	169 (2)

Symmetry codes: (i) *x*, *y*−1, *z*; (ii) −*x*+1, −*y*+1, −*z*.
